# PSICIC: Noise and Asymmetry in Bacterial Division Revealed by Computational Image Analysis at Sub-Pixel Resolution

**DOI:** 10.1371/journal.pcbi.1000233

**Published:** 2008-11-28

**Authors:** Jonathan M. Guberman, Allison Fay, Jonathan Dworkin, Ned S. Wingreen, Zemer Gitai

**Affiliations:** 1 Department of Molecular Biology, Princeton University, Princeton, New Jersey, United States of America; 2 Department of Microbiology, College of Physicians and Surgeons, Columbia University, New York, New York, United States of America; Ben Gurion University, Israel

## Abstract

Live-cell imaging by light microscopy has demonstrated that all cells are spatially and temporally organized. Quantitative, computational image analysis is an important part of cellular imaging, providing both enriched information about individual cell properties and the ability to analyze large datasets. However, such studies are often limited by the small size and variable shape of objects of interest. Here, we address two outstanding problems in bacterial cell division by developing a generally applicable, standardized, and modular software suite termed Projected System of Internal Coordinates from Interpolated Contours (PSICIC) that solves common problems in image quantitation. PSICIC implements interpolated-contour analysis for accurate and precise determination of cell borders and automatically generates internal coordinate systems that are superimposable regardless of cell geometry. We have used PSICIC to establish that the cell-fate determinant, SpoIIE, is asymmetrically localized during *Bacillus subtilis* sporulation, thereby demonstrating the ability of PSICIC to discern protein localization features at sub-pixel scales. We also used PSICIC to examine the accuracy of cell division in *Esherichia coli* and found a new role for the Min system in regulating division-site placement throughout the cell length, but only prior to the initiation of cell constriction. These results extend our understanding of the regulation of both asymmetry and accuracy in bacterial division while demonstrating the general applicability of PSICIC as a computational approach for quantitative, high-throughput analysis of cellular images.

## Author Summary

Recent studies have shown that all cells, including bacteria, are highly spatially organized. However, many questions about bacterial organization remain unanswered, often due to difficulties associated with visualizing and analyzing structures within such small and variably shaped cells. We have overcome these limitations by developing a generally applicable computational method for quantitatively analyzing cellular images at subpixel resolution. Our method uses interpolation to find cell borders accurately and precisely. Using these contours as a starting point, we automated the construction of a general-purpose internal coordinate system for each cell to facilitate comparisons between differently shaped cells. We applied this new method to two unsolved problems in bacterial cell biology. We first showed that a *Bacillus subtilis* asymmetric-division regulator is itself asymmetrically localized, thereby demonstrating our ability to extract information previously thought inaccessible by light microscopy. We also demonstrated our newfound ability to study characteristics of large populations by studying the accuracy of the symmetric division of *Escherichia coli*. We discovered a new role for the Min system, which inhibits polar division, in regulating division throughout the cell length. These results deepen our understanding of two important problems in bacterial cell biology while demonstrating the utility of our approach to studying subcellular structure in a wide range of biological systems.

## Introduction

Biological light microscopy has been pushed to remarkable limits of resolution, speed, throughput, and ease by advances in protein-labeling methods, sample preparation, and microscopy hardware. This imaging has revealed that the subcellular environments of cells from all kingdoms are exquisitely organized both spatially and temporally. Quantitative analysis of such images to measure cell morphologies and track subcellular components in space and time extends the power of cellular imaging by enabling both the extraction of subtle, non-obvious information and the automatic characterization of large datasets. The obvious power of quantitative analysis has prompted many groups to implement ad-hoc, often labor-intensive methods for analyzing specific aspects of their images of interest [1],[2]. However, quantitation of cellular data from light microscopy poses two significant challenges. The first challenge is to identify cell borders with high accuracy and precision despite the limitations imposed by the diffraction of light and the relatively small size of many cells of interest, particularly bacteria. The most common method of identifying cell outlines, thresholding, produces jagged edges that do not accurately represent the smooth shapes of most cells [1]. Recent work has moved beyond this method, utilizing interpolation to increase spatial resolution and define more accurate cell borders [3]–[5]. We have automated this method in a generally-applicable fashion, using interpolated contours to define cell borders with nearly an order of magnitude greater accuracy and precision than traditional approaches. The second challenge is to meaningfully compare different cells within a population despite the presence of complex cellular geometries and natural variations in cell size and shape. We have devised a novel computational methodology to address this challenge, establishing an internal coordinate system for each individual cell that can be readily superimposed to facilitate comparisons among cells. These methodologies have been packaged into a software suite we term Projected System of Internal Coordinates from Interpolated Contours (PSICIC). The modularity, generality, and high-throughput automation of the PSICIC toolkit are such that it can be applied to virtually any type of cell in order to measure many different parameters of shape and localization.

Cellular imaging studies from the past decade have clearly demonstrated that bacterial cells, like their eukaryotic counterparts, are spatially and temporally organized [6],[7]. Bacteria afford many experimental advantages as model cells, including ease of experimental manipulation and the ability to image large numbers of cells. Balancing these advantages, the power of bacterial cell biology is limited by the small size of bacteria, which is on the same scale as the wavelengths of light used for imaging. The variable shapes of many bacterial species presents an additional complication for quantitatively analyzing images of bacterial morphology and protein localization. PSICIC's strengths in extraction of highly accurate spatial data and insensitivity to variable cell borders directly address these limitations. As a demonstration we have applied PSICIC to two important problems in bacterial cell biology: asymmetric protein localization in *Bacillus subtilis* sporulation, and the accuracy of cell-division-site placement in *Escherichia coli* cytokinesis.

*B. subtilis* sporulation involves an asymmetric cell division event that gives rise to a larger mother cell and a smaller forespore [8],[9]. SpoIIE is a membrane-bound phosphatase that contributes to the asymmetric differentiation of these two cells by selectively activating the σ^F^ transcription factor in the forespore [10]. Though the biochemical mechanism of σ^F^ activation by SpoIIE is understood, the basis for the preferential activity of SpoIIE in the forespore compartment remains unclear. A recent study used genetic arguments to suggest that SpoIIE is preferentially localized to the forespore face of the sporulation division septum [11]. However, conventional image analysis of SpoIIE localization could not detect this asymmetry [12]. Here, we used PSICIC to quantitate SpoIIE localization with high accuracy and precision and directly established that SpoIIE is asymmetrically targeted to the forespore face of the sporulation division plane. These results establish the ability of PSICIC to extract subtle yet biologically significant protein localization features from conventional light microscopy at scales that were once thought to be exclusive to far more labor-intensive methods such as immuno-electron microscopy.

Cell division in *E. coli*, unlike *B. subtilis* sporulation, is a symmetric process that results in two similarly-sized daughters [13],[14]. While there are two systems known to contribute to division accuracy, the Min system and nucleoid occlusion [15],[16], where and when they act within the cell and how much each contributes to division accuracy is not well understood. For example, the Min system is known to block erroneous polar divisions [16], but the extent to which it contributes to the accuracy of symmetric division remains unclear. Experimental and theoretical studies have given contradictory results and predictions [13],[17],[18]. By exploiting the ease of large-scale automated analysis with PSICIC, we directly measured the accuracy of midcell division with unprecedented data density. We also quantitated the nature of division in a mutant that lacks the Min system. Our data demonstrate that *E. coli* divides with extreme accuracy, and that the Min system contributes to this accuracy by doing more than simply blocking polar divisions. We also find that in both wild-type cells and cells lacking a functional Min system, the division site is accurately chosen before the cell begins to constrict and the accuracy of division-site placement is not significantly improved after a division pinch appears.

In addition to contributing to the understanding of sporulation and division, our analyses of SpoIIE localization and division-site accuracy are intended to highlight multiple aspects of the power of PSICIC in analyzing data from different species, extracting single-cell and population statistics, and utilizing both morphological and protein localization information.

## Results

### Rationale and Implementation of PSICIC

**Figure 1:**
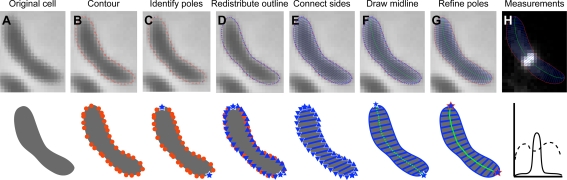
Schematic view of the implementation of PSICIC. (A) The original image, prior to analysis. (B) The set of points (red dots) at which the image intensity crosses a given threshold is calculated, defining a contour for that cell. The given points are unevenly distributed. (C) The pair of points (stars) on the contour that are the greatest Euclidean distance apart are chosen as a first approximation of the poles. The choice of poles divides the contour into two curves (called “left” and “right” for simplicity). (D) An equal number of points (blue triangles) are evenly distributed along the left and the right curves, such that the distances between points on the left curve are all equal, but not necessarily equal to the distances between points on the right curve. (E) Each point on the left curve is paired with the corresponding point on the right curve, and a straight line, called a “width line” (blue lines) is drawn connecting the pair. (F) The midline (dotted green line) is drawn through the midpoint of each of the width lines. (G) Each pole is moved stepwise and the process described above iterated until the longest midline is identified (solid green line). (H) Using the resulting internal coordinate system of midline and width lines, measurements, such as cell width (dashed line) or fluorescence intensity (solid line), can be quantified.

To achieve robust and accurate quantitation, PSICIC sequentially applies two approaches to address two different imaging problems: interpolation to more precisely identify cell borders, and establishment of internal coordinate systems to enable direct comparisons among cells. When analyzing a digital image, the first task is to identify the regions of interest. In biological applications, this often amounts to defining the cell borders. In phase microscopy, cells appear as dark objects on a light background (Figure 1A). Therefore, the simplest and most common way to identify cells in a phase micrograph is by setting a binary threshold value: pixels darker than a given threshold are flagged as being inside a cell, and pixels lighter than that threshold are considered outside of the cell. Regions of adjacent pixels marked as inside a cell are grouped together and the resulting set of pixels can then be further analyzed as a group. While this thresholding method can reliably identify pixels that are either fully inside or fully outside a cell, it does not deal well with pixels that span a cell border, resulting in jagged borders. From electron microscopy we know that cell outlines are smooth on the scale of light microscopy, and therefore the pixilated borders produced by the thresholding method are inaccurate representations of the cell. This limitation becomes problematic when trying to make measurements on the scale of single pixel sizes, as is often necessary for small cells like bacteria.

To overcome the problem of jagged borders caused by thresholding, our method uses interpolated contours to define the outline of a cell (Figure 1A and 1B). Generating smooth contours from a grid of values is a standard problem in which the interpolation of values between the grid positions is used to define where the image crosses a given intensity threshold [19]. In our implementation, the initial choice of the intensity threshold at which to draw the cell outline is arbitrary, but the threshold value is then optimized to yield the contour for which the intensity change between the cell and the background is most drastic. This is accomplished by finding the threshold where the total area enclosed by the contour is the least sensitive to small changes in the threshold value. The contours chosen in this manner agree well with methods that require calibration of the images against a membrane dye [3],[4], and have the added advantage that they do not require system-specific calibration. This method for choosing the intensity threshold at which to draw the contour has the additional advantages of not relying on any global parameters of the image and of being insensitive to the contrast between the cell and the background. However, for applications for which a specific threshold is desired, PSICIC can use any given value as the threshold.

At this stage in the analysis, having identified the regions of interest in the image, objects can optionally be filtered out by any number of criteria to remove false positives (such as dirt on the slide) or other undesirable objects (such as cells clumped together). This filtering can be done on the basis of size, shape, or more sophisticated metrics such as the smoothness or curvature of the cell outline.

In order to measure the location of objects within the cell, and to be able to compare these measurements between cells that may vary in shape and size, we use the interpolated contours that define the cell outlines to create an internal coordinate system for each cell. This allows measurement of the location, size, and shape of subcellular features, such as the localization of fluorescently-labeled proteins, relative to the geometry of each specific cell. The cells that we study are generally rod-shaped or variants on a rod shape, which suggests the length of the cell as a natural axis from which to begin measurements. PSICIC therefore establishes a projected internal coordinate system by finding a midline connecting the poles of the cell as the long axis, and non-intersecting lines through this midline that locally define the other axis (Figure 1C–1G). The first step in this process is the identification of the poles of the cell, which are initially approximated by the two points on the border of the cell most distant from each other (Figure 1C). An equal number of points are then evenly distributed between the two poles along both the left and right halves of the cell contour (Figure 1D). These points help generate a cell midline: we connect each pair of left and right points to establish width lines, find the midpoints of each width line, and define the midline as a line that connects all of those midpoints (Figure 1E and 1F). By iteratively applying this procedure, the cell poles are redefined as the pair of points separated by the longest midline, thereby accommodating cell shapes for which the poles are not the two most distant points, such as crescents (Figure 1G). The final midline and width lines uniquely identify the projected position of any point in or on the cell, and therefore represent an internal coordinate system. Once generated using a phase image, this internal coordinate system acts as a digital representation of the cell, which can be overlaid on other channels of a micrograph to measure the intensity and location of a fluorescent marker or other properties of the cell, such as the position and magnitude of the division invagination (Figure 1H). The length and shape of the midline, as well as the lengths of the width lines, can also be used to further quantify the shape of the cell.

### PSICIC Measurements Are Accurate and Precise

In order to validate the measurements generated by PSICIC, we tested both its precision and accuracy. Precision was tested computationally by performing measurements on simulated images of known dimensions. Accuracy was tested using micrographs of beads whose dimensions had been independently verified by electron microscopy.

**Figure 2:**
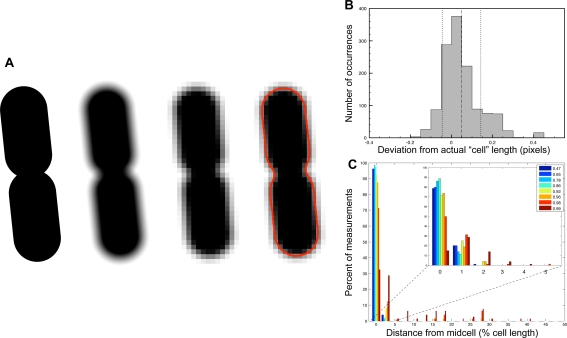
*In silico* tests of PSICIC precision. (A) Symmetrical cell shapes were generated (first cell from left), rotated to different angles (not shown), blurred to simulate the point-spread function of our microscope (second cell), pixilated at a spatial density similar to that of the microscope (third cell), and measured by PSICIC (fourth cell). (B) Distribution of the difference between actual “cell” length and length measured by PSICIC, measured in pixels. The dashed line shows the mean deviation (+0.049 pixels, equivalent to 6.3 nm for the imaging apparatus used for the subsequent *E. coli* division experiments) and the two dotted lines show plus and minus one standard deviation (±0.094 pixels, equivalent to 12.2 nm). (C) The deviation of measured division-site location from midcell in symmetrically pinched “cell” images, as a percentage of cell length. Colored bars represent different pinch depths, measured by the thickness at the pinch as a fraction of cell thickness away from the pinch. Inset shows detailed data for the 5% of cell length closest to midcell.

To test that the measurements used by PSICIC to generate a digital representation of a cell are precise, and that no systematic bias is introduced in the measurement process, we used PSICIC to analyze a set of simulated cells of exact known dimensions (Figure 2). In this *in silico* experiment, the digital representation of various cells was manipulated to reflect the process of digital imaging: cells were rotated to several different angles to simulate different orientations with respect to a fixed pixel grid, and then blurred to simulate light scattering using a point-spread function similar to that of the imaging apparatus used for the subsequent experiments in this study. Each resulting object was then pixilated by overlaying it with a grid with spacing equivalent to the pixel size used for subsequent data acquisition and assigning each pixel the value of the sum of the intensities of the points within the corresponding grid square (Figure 2A). PSICIC was used to analyze the resulting simulated image, and measurements of cell width, length, and area were compared to those of the original unprocessed image. Using this *in silico* approach, we examined 80 cells, each rotated 16 times. We found that the difference between the expected and measured length had a standard deviation of 0.0617 pixels, which is the equivalent of 8 nanometers in an image taken from our 100× phase objective (Figure 2B). PSICIC thus provides a greater than 15-fold improvement in precision relative to traditional single-pixel-limited approaches.

**Figure 3:**
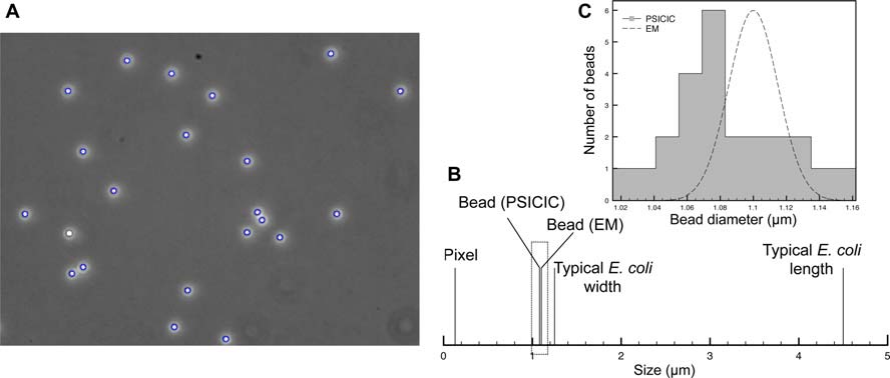
Measurement of beads of known size. (A) Phase contrast image of 1 µm diameter beads (100× magnification) with PSICIC identification of outlines overlaid (blue lines). (B) Comparison of the size in microns of: a pixel in these images, mean bead size measured by PSICIC, mean bead size measured by electron microscopy, typical *E. coli* width, and typical *E. coli* length. (C) The distribution of bead sizes as measured by PSICIC (gray bars) compared to the expected distribution obtained from electron microscopy data (dashed curve).

Though PSICIC can produce an intensity value at arbitrary image resolution, the measurements will only be valid up to a certain level of accuracy, determined as a fraction of the pixel size of the image. To assess the accuracy of PSICIC in measuring real-world objects, we used the software to measure the diameters of beads of tightly controlled size (Figure 3). The manufacturer's measurement, performed by electron microscopy, for the specific batch of beads analyzed gave a distribution of diameters with mean 1.1 µm and standard deviation (S.D.) 0.035 µm. We imaged these beads by light microscopy using a 100× 1.4 NA phase oil objective. PSICIC measurements of these light microscopy images yielded an average bead diameter of 1.08 µm and S.D. 0.030 µm (Figure 3B). The images have a pixel size of 0.13 µm, so the 0.02 µm difference in estimated mean diameter suggests that PSICIC is able to achieve a more than six-fold gain in spatial accuracy over pixel-limited methods. The ability of PSICIC to accurately measure the bead size, despite the fact that the beads differ significantly in size and refractive index from cells, validates the robustness of the choice of contour, and the adaptability of PSICIC to a wide variety of data types. Together, these results validate the use of PSICIC to extract sub-pixel high-resolution data on cells from light microscopy images.

### PSICIC Detects Asymmetries in Protein Localization during *B. subtilis* Sporulation

The accuracy obtainable using PSICIC can reveal phenotypes that are not obvious by visual inspection. To illustrate this ability, we examined the localization of the membrane phosphatase SpoIIE during *B. subtilis* sporulation. SpoIIE asymmetrically activates the sporulation sigma factor σ^F^ in the forespore, thereby contributing to the establishment of polarity in sporulation [10]. Initially, SpoIIE is expressed pre-divisionally and then localizes to the division septum that separates the mother cell and the forespore. Though SpoIIE activity is asymmetric, conventional image analysis failed to detect any asymmetry in SpoIIE localization at the sporulation septum [12].

**Figure 4:**
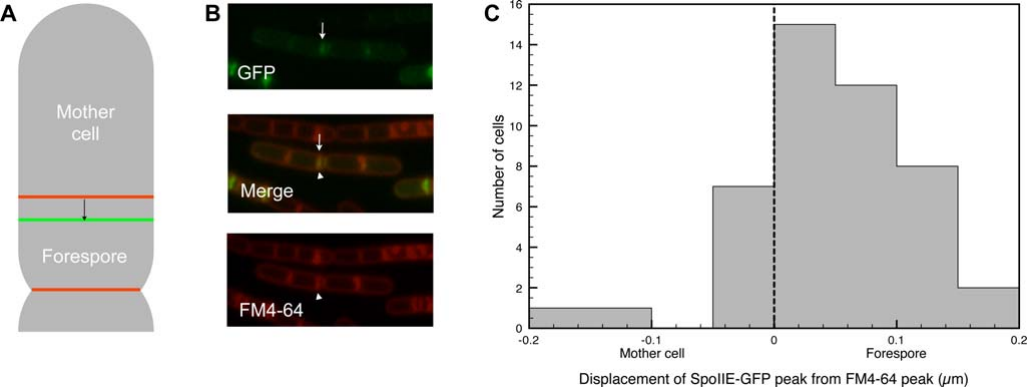
SpoIIE-GFP is preferentially localized to the forespore in *B. subtilis*. (A) Schematic showing the displacement of SpoIIE-GFP (green) from FM4-64 (red), and the displacement measured by PSICIC (arrow). (B) A typical *B. subtilis* image, showing the GFP channel (top), FM4-64 channel (bottom), and merged image (middle). Highlighted are a SpoIIE-GFP peak (arrow) and an FM4-64 peak (arrowhead). (C) Histogram showing the magnitude and direction of SpoIIE-GFP displacement towards (positive) or away from (negative) the forespore.

To directly examine whether SpoIIE is asymmetrically localized during sporulation, we used PSICIC to extract sub-pixel information and determine the distribution of SpoIIE-GFP with respect to the early asymmetric septum (Figure 4). Specifically, we imaged cells bearing a functional SpoIIE-GFP fusion that is driven by the endogenous SpoIIE promoter and that replaces the wild-type copy of the protein [20]. These cells were also stained with the red membrane dye FM4-64 to visualize the sporulation septum. The locations of the peaks in each fluorescent channel were obtained for each analyzed cell by summing the fluorescence intensity along each width line of the cell's internal coordinate system to generate a single-dimensional intensity projection onto the midline. Reducing the data to one dimension aids in analysis, and summing the data rather than examining a slice of the cell helps improve the signal-to-noise ratio. To identify the peaks of these intensity projections, local maxima were identified and the first moment of the intensity in a region around each maximum was calculated. This first-moment method gives a result that is more robust to noise than simply recording the location of the peak, and has the further advantage of appropriately weighting any asymmetries in the distribution of fluorescence. Because *B. subtilis* forms chains of unseparated cells, the location of measurable division septa was used to delineate individual cells and to distinguish sporulation septa from vegetative septa.

The peaks of the SpoIIE-GFP and FM4-64 intensities were measured and compared in 46 sporulating cells. In 38 of the 46 cells (83%), the SpoIIE-GFP peak was shifted towards the forespore daughter cell, while 6 cells (13%) had a SpoIIE-GFP peak shifted towards the mother cell, and two cells (4%) had SpoIIE and FM4-64 peaks that colocalized to within the limits of PSICIC's measurement capabilities (Figure 4C). The mean distance between the septum and SpoIIE signal was shifted 63±54 nm towards the forespore. As a control for systematic measurement errors, we examined cells whose membranes were co-stained with red FM4-64 and a green nonyl acridine orange (NAO) dye that binds negatively charged lipids [21]. Relative to the FM4-64 peaks, these cells exhibited an average NAO peak displacement of 0.1±55 away from the forespore. The statistically significant difference between the SpoIIE displacement and the dual-membrane labeling displacement (P = 0.016) indicates that SpoIIE-GFP localization is indeed asymmetric and biased towards the forespore side of the septum. It is unclear whether the 6 (out of 46) cells displaced towards the mother cell were due to measurement error or biological variability. Our dataset included several chains of cells with multiple sporulation septa that exhibited displacement in opposite absolute directions, but with both being displaced towards the forespore, further supporting the conclusion that the measured forespore-oriented asymmetry of SpoIIE-GFP localization is not due to error in our data or analysis. Together, these results indicate that SpoIIE is preferentially localized to the forespore side of the sporulation septum and that this previously undescribed asymmetry can be detected by using PSICIC to analyze conventional light microscopy images.

### Probing the Function of the Min System through PSICIC Analysis of the Accuracy of Cell Division in *E. coli*

The automated nature of PSICIC lends itself to the analysis of large numbers of cells to measure distributions of morphological characteristics as well as noise and accuracy of subcellular processes. One such process whose accuracy is actively regulated is division-site placement in *E. coli*, which is highly symmetric in wild-type cells [13],[14]. The active regulation of division accuracy suggests that equal division has a significant impact on cellular fitness. *E. coli* cells have evolved at least two distinct mechanisms that improve the accuracy of division-site placement: the Min system, which prevents division near the cell poles, and nucleoid occlusion, which prevents division from occurring over the nucleoid [15],[16]. The Min system is the better-characterized of these mechanisms [22]. MinC is a negative regulator of the assembly of the cell-division protein FtsZ [23]. MinC binds to the MinD ATPase on the membrane [24], and MinE regulates the ATP hydrolysis and cooperative assembly of MinD to produce a stable pole-to-pole oscillation [25],[26]. The result of this oscillation is that the time-averaged concentration of MinC is highest at the cell poles [27], thereby biasing FtsZ assembly away from the cell ends and preventing polar divisions that would produce inviable minicells. While the role of the Min system in preventing minicelling is clear, whether Min acts throughout the cell length or merely in the polar regions and whether Min acts solely before division begins or throughout cytokinesis has remained unclear [13],[17],[18].

**Figure 5:**
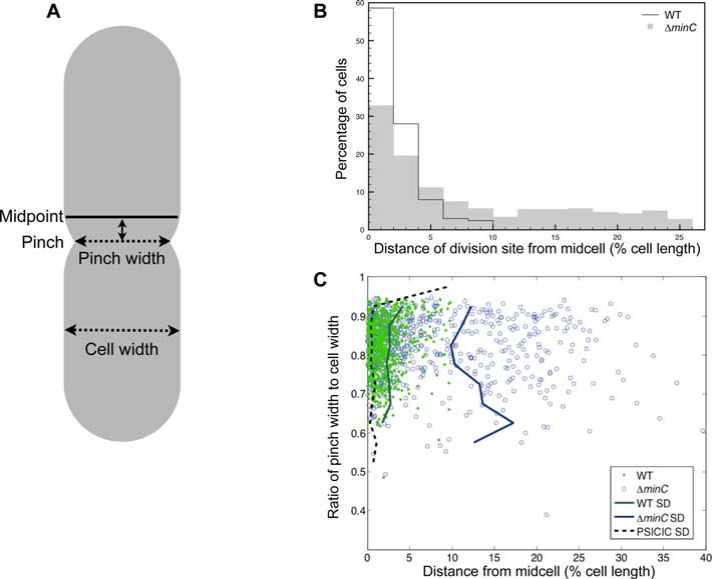
Pinch position measurements in *E. coli*. (A) Schematic showing an asymmetrically dividing cell, indicating the geometric midpoint of the cell (solid line), the pinch position and width, the distance of the pinch position from midcell (double-headed solid arrow), and the maximal cell width. (B) Distribution of the distance of the pinch position from midcell, as a percentage of cell length, shown for wild-type (black line) and Δ*minC* (gray shading) strains. (C) Scatter plot of pinch position versus the depth of the pinch for wild type (green dots) and Δ*minC* (blue circles). Standard deviation is shown for wild type (solid green curve), Δ*minC* (solid blue curve), and the theoretical limits of PSICIC (dashed black curve, see also Figure 2C).

To determine the accuracy with which *E. coli* divides and the role of the Min system in achieving this accuracy, we used PSICIC to analyze the pinch position of over 3,000 wild-type and *minC* mutant cells (Figure 5). We found that wild-type cells divide with high accuracy with a S.D. of only 2.9% of cell length. This result is validated by its strong similarity to that of a previous study that used the far more labor-intensive approach of electron microscopy to analyze fixed cells and yielded 2.5% S.D. [14]. To determine the accuracy limits of our measurements we used the *in silico* approach described above to examine simulated cells with exactly symmetric pinch positions of different depths (Figure 2C). We found that PSICIC could identify the location of even extremely subtle, 8% pinch depths with better accuracy than we observed in any experiments (0.64% S.D.), indicating that the observed noise in Figure 5 is dominated by biological variability rather than measurement noise.

In light of their frequent polar divisions, it was not surprising to find that *minC* mutants had significantly reduced division-site-placement accuracy (11.5% S.D.). Interestingly, however, when we limited the analysis to cells that divided within the central half of the cell, *minC* mutants still exhibited significantly (P = 1.3e-53) worse division-site-placement accuracy (8.2% S.D.) compared to wild type (2.9% S.D.) (Figure 4B). These results suggest that in addition to its role in preventing polar divisions, the *E. coli* Min system regulates division-site placement throughout the cell length, including near midcell.

By examining cells with different extents of division-plane constriction (pinch depth), we were also able to analyze the nature of the division process itself. We found that in both wild-type and *minC* cells, the accuracy of cell division does not significantly correlate with pinch depth (Figure 4C). This result indicates that division-site placement is fixed before or soon after constriction begins, and that once division initiates, the accuracy of the division site is not further refined. We also examined the shape of each cell constriction by measuring the ratio of the pinch depth to the pinch width. The pinch shapes of wild-type and *minC* mutants were similar and remained relatively unchanged over a wide range of pinch depths (data not shown). This result is consistent with the conclusion that the Min system does not function during the division process and supports a model wherein the division site is fixed before constriction begins. These findings thus suggest that the Min system acts throughout the cell length to regulate division-site accuracy prior to the initiation of cell constriction but does not participate in the division process itself.

## Discussion

Advances in fluorescent protein labeling, sample preparation, and image acquisition have pushed light microscopy to its physical limits. Techniques such as deconvolution, photo-activated localization microscopy (PALM), and stochastic optical reconstruction microscopy (STORM) have increased spatial resolution further still [28],[29]. However, analysis of light microscopy images has rarely taken advantage of the knowledge that cell boundaries are smooth well below the wavelength resolution of light or implemented approaches for comparing cells of variable and irregular morphologies. Here we introduced PSICIC as a robust and generally-applicable computational method for automating the high-resolution quantitative analysis of cellular image data. We demonstrated that PSICIC can identify cell borders with accuracy and precision nearly an order of magnitude greater than conventional pixel-limited thresholding approaches, bringing the information content of light microscopy images towards the nanometer-scale regime once thought to be exclusive to electron microscopy. While techniques such as PALM and STORM increase the effective resolution of fluorescent microscopy, they require multiple images of fixed cells [28],[29]; PSICIC can be used on any images, including live cell cultures, and can be combined with deconvolution techniques for even greater gains in analytical power. In addition to creating reproducible measurements and increasing spatial precision, the automated nature of PSICIC enables rapid gathering of large quantities of data. Measuring more cells allows the use of powerful statistical tools to analyze the data. Furthermore, the establishment of an internal coordinate system by PSICIC enables direct comparison of the localization of subcellular features between cells that may differ in size and shape. Recent work has shown that even rod-shape bacteria, such as *E. coli*, exhibit asymmetrical shapes, supporting the usefulness of an internal coordinate system which takes this asymmetry into account for studies of cell shape and localization [3]. The internal coordinate system generated by PSICIC facilitates studying both the variation within a population of cells, and the variation between different populations of cells. Studying variation within a population gives insight into how noisy a system is, and can reveal how tightly regulated a process is, either in time or in space. When comparing different populations, obtaining more data on each population can reveal subtle differences in phenotype that might otherwise have been overlooked. The automated analysis of large quantities of imaging data could also be useful in large-scale, genome-wide studies of shape and localization.

In this study we exploited the ability of PSICIC to analyze single cells with great precision and obtained new insights into both symmetric and asymmetric bacterial division. We established that the cell-fate determinant SpoIIE is preferentially targeted to the forespore side of the division plane during the asymmetric division of *B. subtilis* sporulation. This result is validated by a recent study demonstrating that during the engulfment phase of sporulation, SpoIIE localization depends on the forespore-specific SpoIIQ protein [11]. Consistent with the hypothesis that SpoIIE becomes more asymmetric during sporulation, our PSICIC analysis found that the extent of SpoIIE's asymmetric displacement towards the forespore increased as sporulation progressed and the sporulation septum grew more curved (data not shown). The ability of PSICIC to directly visualize an asymmetrical protein localization previously hypothesized on genetic grounds demonstrates the power of PSICIC to reveal subtle yet biologically relevant information about protein localization. This result also suggests that in many cases conventional light micrographs contain more spatial information than was previously appreciated, and that proper analysis of this information can often circumvent the need for labor-intensive fixed-cell imaging methods such as electron microscopy or sub-diffraction-limited light microscopy.

In addition to addressing single-cell properties such as SpoIIE asymmetry, we also applied PSICIC to population properties such as the accuracy of the symmetric division of *E. coli*. Despite the fact that *E. coli* division has been intensely studied before, the resolution and scale of our PSICIC analysis yielded new insights. Specifically, we found that division accuracy is tightly regulated by the Min system throughout the cell length prior to the initiation of cell constriction, but that once division starts, it proceeds through a constant and highly stereotyped process that is apparently immune to the influence of Min. While our data are strikingly similar to electron microscopy-based measurements of *E. coli* cell-division accuracy [14], the division accuracy that we report here is somewhat less than the previously-reported accuracy of the localization of FtsZ, the master regulator of *E. coli* division [13]. Our conclusions on the role of Min throughout the cell length also differ from the conclusions of a recent study of the Min system in germinating *B. subtilis* cells that proposed that Min does not regulate midcell division-site placement [18]. It is possible that FtsZ localization does not perfectly correlate with cell-division localization in *E. coli* and that the Min systems of *E. coli* and *B. subtilis* differ. Alternatively, the traditional analyses used by the prior studies did not address pixilation effects, implying that the data is inherently binned. For example, if one uses 100 nm pixels, all divisions within 100 nm are reported as perfectly accurate, thereby generating an overestimate of division accuracy and obscuring subtle differences between populations.

The modular nature of PSICIC allows for wide expandability and utility. Once the data has been analyzed and the digital cell constructed, measurements of any sort can be performed, such as morphometrics or fluorescence intensity in either one or two dimensions. The high-throughput capacity allows for screens based on shape or localization phenotypes. Given a series of time-lapse images, individual cells can also be tracked over time in order to observe dynamic changes in shape or localization. In this study we exploited the flexibility of PSICIC to analyze multiple properties of two different bacterial species imaged on two different apparatuses. Though PSICIC was created with bacterial cells in mind, it can easily be applied to both unicellular and multicellular eukaryotic systems and could be readily modified to create a pseudo-radial, rather than pseudo-Cartesian, internal coordinate system for analyzing round cells that lack a clear major axis. Computational analysis of fluorescent microscopy offers many exciting possibilities that will only increase as modern imaging techniques are used and expanded by the research community. We have thus made available the source code, software, and documentation for PSICIC (see link at http://www.molbio1.princeton.edu/labs/gitai/), and encourage others to modify, expand, and adapt the software capabilities to suit their own applications.

## Materials and Methods

### Calculation and Choice of Contours

Contours were calculated using the MATLAB function contourc, described in “The Contouring Algorithm” [19]. Briefly, the intensity value of the image at any arbitrary point is estimated using the values of the pixels nearest to that spatial point, using a distance-weighted average of the surrounding. The function contourc returns ordered sets of points for which the interpolated value of the image intensity is equal to the contour intensity level given as input. When these sets of points are connected in order by straight line segments, the result is a closed polygon which approximates a smooth curve defining the border of the object.

The initial choice of contour intensity level was determined by manually choosing a value which produced contours around the objects of interest that were well-separated such that the borders of nearby cells did not intersect. The choice of level was then refined on an automated cell-by-cell basis by generating contours for a range of different levels centered around the original value. The same object was identified in each set of contours by matching the centers of mass between contours at each level. The derivative of cell area with respect to contour intensity level was then calculated for each cell, and the contour level at which the minimum derivative occurs was used as the final contour for that particular cell.

### Establishing an Internal Coordinate System

In order to define an internal coordinate system for a cell, the cell poles must first be identified. The pair of points on the outline of the cell that are farthest away from each other are used as the first approximation of the poles, an estimate that is refined later. Having defined the poles, the cell outline is partitioned into two curves, one running clockwise from one pole to the other pole, the other counterclockwise. These will be referred to as the left and right halves of the curve (though the nomenclature is arbitrary). The left and right curves are then subdivided into *n* points each, which are equally spaced along each half (though the spacing for the left half need not equal that of the right; the spacing is determined by the arc length of each curve). The number of points, *n*, can be chosen arbitrarily, but should be chosen to over-sample the original data; if *n* is too small, data will be missed, but if *n* is too large, the computation time increases significantly. For the studies reported in this work, *n* = 200 was found to sufficiently oversample to capture the variation within the data set (data not shown). Each point on the left curve is then connected to the corresponding point on the right curve, resulting in a set of n non-intersecting lines, called the width lines. The midpoints of the width lines are connected to create the midline of the cell. The final step in this process is to optimize the choice of the poles of the cell by moving the poles stepwise along the original contour and repeating the entire process to determine the length of the midline generated. If the new midline is longer than the last, the search continues in that direction; otherwise, the search terminates. This search is performed four times, both clockwise and counterclockwise, starting at both poles, and the longest of the four midlines generated is used. The longest midline together with the set of width lines then forms the coordinate system.

### Filtering Data

The data can be filtered by any number of criteria. Establishing a minimum area and/or perimeter for objects helps to filter dirt from the images; establishing a maximum area is often necessary to prevent large regions of uneven coloration on the slides from being identified as cells. To filter out cells that are adjacent to one another and cannot therefore be individually identified, filtering based on width was found to be the most effective method (data not shown). The original images overlaid with the cell borders identified by PSICIC were manually verified to check for false positives.

### Computational Analysis of PSICIC Precision

Simulated images of 16 symmetrically dividing cells were created in Keynote (Apple). The original images varied in length from 474 pixels to 716 pixels, and pinch depth varied from fully divided to barely pinched (thickness at the pinch 98% that of the rest of the cell). Each of the images was blurred using the MATLAB function imfilter, using a disk filter generated by the fspecial function with a radius of 20 pixels, in order to simulate the point spread function of the microscope. Each of the blurred cells was then rotated to 16 different orientations, to account for any effect of angle on PSICIC measurements. The 256 blurred and rotated cells were each then binned by a factor of 5, 10, 15, 20, and 25 to generate 1280 cells with different extents of pixilation. The cell lengths and pinch positions were then analyzed using PSICIC, and the measurement in pixels was multiplied by the magnification factor of that image for comparison with the known length of the original unprocessed cell image. The position of the pinch was also measured as a percentage of the measured cell length and compared to the known pinch position of exactly 50% of cell length.

### Microscopy of Beads

Molecular Probes FluoSpheres carboxylate-modified microspheres, 1.0 µm, yellow-green fluorescent beads were imaged on 1% agarose pads in water using a QImaging Rolera-XR camera on a Nikon 90i microscope with a Nikon Plan Apo 1.4/100× Oil Ph3 phase objective, using the NIS Elements software package. The images were then analyzed using PSICIC without midline optimization, and the diameter of the beads calculated by multiplying the measured length in pixels with the known pixel size of the microscope objective (0.13 µm/pixel). The measured length was compared to the length measured by transmission electron microscopy that was reported on the certificate of analysis for the specific lot of beads analyzed (lot number 45102), given as 1.1±0.035 µm.

### Analysis of SpoIIE Localization during *B. subtilis* Sporulation

Cultures of PY79 and Jdb872 (SB201) *spoIIE*-gfp kan [20] were sporulated by resuspension. At 60 minutes after resuspension, 100 µl of sporulating cells were taken. To Jdb872, 0.5 µl of FM4-64 (Invitrogen; 100 mg/ml) was added just before the cells were collected by centrifugation. To PY79, 0.5 µl of FM4-64 (100 mg/ml) and 1.0 µl nonyl acridine orange (Invitrogen; 10 nM) were added. The pellet was resuspended in 10 µl PBS and added to a poly-L-lysine pre-treated coverslip. The cells were imaged using a Nikon Eclipse 90i with a 100× objective using phase contrast and captured by a Hamamatsu Orca-ER camera using Nikon Elements BR software. Exposure for FITC and TRITC was 300 ms for all pictures taken.

Images were analyzed by PSICIC. For each fluorescent channel a one-dimensional intensity profile along the midline of the cell was generated by taking the sum of the interpolated intensities at 20 evenly-spaced points along each width line. The location of peaks in the intensity data was calculated by first finding local maxima in the intensity profile. In order to account for asymmetry in the shapes of these peaks, the points where the intensity crossed a threshold of 90% the peak value on either side of the local maxima were found, and the first moment of the intensity between those points was taken. Specifically, the point marked as the peak is given by
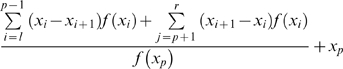
where *x_i_* are the points along the midline, *x_p_* is the initially located peak, *x_l_* and *x_r_* are the 90%-maximal-intensity boundaries to either side of the peak, and *f(x)* is the value of the intensity profile at the point *x* along the midline.

The distance between peaks of SpoIIE-GFP and FM4-64 intensity was calculated for adjacent peaks located within one quarter-cell length of the end of a cell (either a cell pole or a cellular division site, defined as a local minimum in the thickness of a cell). Peaks not significantly higher than the background fluorescence level, peaks distant from the end of a cell, and peaks with no corresponding nearby peak in the other fluorescent channel were ignored. The sign of the distance between peaks was given as positive if the SpoIIE-GFP peak was closer to the nearby cell end, and negative if the FM4-64 peak was closer to the nearby cell end.

### Analysis of *E. coli* Division Accuracy

*E. coli* strains BW25113 [30] and Δ*minC::Kan* [31] were grown overnight in LB and LB plus 30 µg/ml kanamycin, respectively, at 37°C. Overnight cultures were diluted 1∶1000 and then grown for an additional 2 hours. Samples were prepared on pads composed of 1% agarose in water, and imaged as described above for the microscopy of beads.

The resulting images were analyzed using PSICIC. To find the pinch position of a cell, the second set of differences between thickness measurements was taken as an approximation of the second derivative for non-continuous data, and smoothed using a two-point moving average to reduce noise. The maximum of the smoothed data was then located, and the local minimum of the thickness nearest that maximum was taken as the pinch position. A pinch was discarded if the depth of the pinch was outside the range reliably identifiable by PSICIC (thicker than 95% of the maximum cell thickness, data not shown). Outliers in both data sets were manually examined to ensure validity.

### Statistical Methods

To determine whether the SpoIIE-GFP displacement data was statistically significantly different from a symmetrical distribution, a two sided *t*-test for a distribution with unknown mean and variance was used [32].

To determine if the variances of the distributions of wild-type and Δ*minC E. coli* were significantly different, the Ansari-Bradley test, a non-paramentric test which does not require data to come from a normal distribution, was used. This test requires both distributions to have the same median, a requirement satisfied by our data sets which both have median 0.5 [19].

### Accession Numbers

The GenBank (http://ww.ncbi.nlm.nih.gov/Genbank) accession numbers for the genes and proteins discussed in this paper are *E. coli* MinC (AAC74260), *E. coli* MinD (AAC74259), *E. coli* MinE (AAC74258), *E. coli* FtsZ (AAC73206), *B. subtilis* SpoIIE (CAB11840), and *B. subtilis* SpoIIQ (CAB15672).

## References

[1] Pincus Z, Theriot JA (2007). Comparison of quantitative methods for cell-shape analysis.. Journal of Microscopy.

[2] Viollier PH, Thanbichler M, McGrath PT, West L, Meewan M (2004). Rapid and sequential movement of individual chromosomal loci to specific subcellular locations during bacterial DNA replication.. Proc Natl Acad Sci U S A.

[3] Itan E, Carmon G, Rabinovitch A, Fishov I, Feingold M (2008). Shape of nonseptated Escherichia coli is asymmetric.. Phys Rev E Stat Nonlin Soft Matter Phys.

[4] Reshes G, Vanounou S, Fishov I, Feingold M (2008). Cell shape dynamics in Escherichia coli.. Biophys J.

[5] Betz T, Koch D, Stuhrmann B, Ehrlicher A, Kas J (2007). Statistical analysis of neuronal growth: edge dynamics and the effect of a focused laser on growth cone motility.. New Journal of Physics.

[6] Gitai Z (2005). The new bacterial cell biology: moving parts and subcellular architecture.. Cell.

[7] Thanbichler M, Shapiro L (2008). Getting organized—how bacterial cells move proteins and DNA.. Nat Rev Microbiol.

[8] Errington J (2003). Regulation of endospore formation in *Bacillus subtilis*.. Nat Rev Microbiol.

[9] Losick R, Dworkin J (1999). Linking asymmetric division to cell fate: teaching an old microbe new tricks.. Genes Dev.

[10] Arigoni F, Duncan L, Alper S, Losick R, Stragier P (1996). SpoIIE governs the phosphorylation state of a protein regulating transcription factor sigma F during sporulation in *Bacillus subtilis*.. Proc Natl Acad Sci U S A.

[11] Campo N, Marquis KA, Rudner DZ (2008). SpoIIQ anchors membrane proteins on both sides of the sporulation septum in Bacillus subtilis.. J Biol Chem.

[12] Levin PA, Losick R, Stragier P, Arigoni F (1997). Localization of the sporulation protein SpoIIE in *Bacillus subtilis* is dependent upon the cell division protein FtsZ.. Mol Microbiol.

[13] Yu XC, Margolin W (1999). FtsZ ring clusters in min and partition mutants: role of both the Min system and the nucleoid in regulating FtsZ ring localization.. Mol Microbiol.

[14] Trueba FJ (1982). On the precision and accuracy achieved by *Escherichia coli* cells at fission about their middle.. Arch Microbiol.

[15] Bernhardt TG, de Boer PA (2005). SlmA, a nucleoid-associated, FtsZ binding protein required for blocking septal ring assembly over Chromosomes in *E. coli*.. Mol Cell.

[16] de Boer PA, Crossley RE, Rothfield LI (1989). A division inhibitor and a topological specificity factor coded for by the minicell locus determine proper placement of the division septum in *E. coli*.. Cell.

[17] Kerr RA, Levine H, Sejnowski TJ, Rappel WJ (2006). Division accuracy in a stochastic model of Min oscillations in Escherichia coli.. Proc Natl Acad Sci U S A.

[18] Migocki MD, Freeman MK, Wake RG, Harry EJ (2002). The Min system is not required for precise placement of the midcell Z ring in Bacillus subtilis.. EMBO Rep.

[19] Mathworks (2007). MATLAB. 2007b ed.

[20] Garti-Levi S, Hazan R, Kain J, Fujita M, Ben-Yehuda S (2008). The FtsEX ABC transporter directs cellular differentiation in *Bacillus subtilis*.. Mol Microbiol.

[21] Kawai F, Shoda M, Harashima R, Sadaie Y, Hara H (2004). Cardiolipin domains in *Bacillus subtilis* marburg membranes.. J Bacteriol.

[22] Lutkenhaus J (2007). Assembly dynamics of the bacterial MinCDE system and spatial regulation of the Z ring.. Annu Rev Biochem.

[23] Hu Z, Mukherjee A, Pichoff S, Lutkenhaus J (1999). The MinC component of the division site selection system in *Escherichia coli* interacts with FtsZ to prevent polymerization.. Proc Natl Acad Sci U S A.

[24] Hu Z, Saez C, Lutkenhaus J (2003). Recruitment of MinC, an inhibitor of Z-ring formation, to the membrane in *Escherichia coli*: role of MinD and MinE.. J Bacteriol.

[25] Hu Z, Lutkenhaus J (2001). Topological regulation of cell division in *E. coli*. spatiotemporal oscillation of MinD requires stimulation of its ATPase by MinE and phospholipid.. Mol Cell.

[26] Raskin DM, de Boer PA (1999). Rapid pole-to-pole oscillation of a protein required for directing division to the middle of *Escherichia coli*.. Proc Natl Acad Sci U S A.

[27] Huang KC, Meir Y, Wingreen NS (2003). Dynamic structures in *Escherichia coli*: spontaneous formation of MinE rings and MinD polar zones.. Proc Natl Acad Sci U S A.

[28] Betzig E, Patterson GH, Sougrat R, Lindwasser OW, Olenych S (2006). Imaging intracellular fluorescent proteins at nanometer resolution.. Science.

[29] Rust MJ, Bates M, Zhuang X (2006). Sub-diffraction-limit imaging by stochastic optical reconstruction microscopy (STORM).. Nat Methods.

[30] Datsenko KA, Wanner BL (2000). One-step inactivation of chromosomal genes in *Escherichia coli* K-12 using PCR products.. Proc Natl Acad Sci U S A.

[31] Baba T, Ara T, Hasegawa M, Takai Y, Okumura Y (2006). Construction of *Escherichia coli* K-12 in-frame, single-gene knockout mutants: the Keio collection.. Mol Syst Biol.

[32] Ross SM (1987). Introduction to probability and statistics for engineers and scientists.

