# Correction: PSICIC: Noise and Asymmetry in Bacterial Division Revealed by Computational Image Analysis at Sub-Pixel Resolution

**DOI:** 10.1371/annotation/1087d9ce-af96-49c7-8074-8da2542cb005

**Published:** 2009-07-10

**Authors:** Jonathan M. Guberman, Allison Fay, Jonathan Dworkin, Ned S. Wingreen, Zemer Gitai

In the Materials and Methods section, there was an error in the equation in the subsection "Analysis of SpoIIE Localization during B. subtilis Sporulation." Please view the correct equation and accompanying text here: 

Specifically, the point marked as the peak is given by


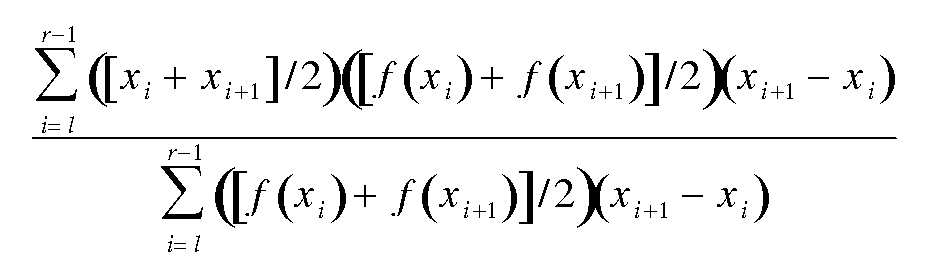


where *x_i_* are the points along the midline, *x_l_* and *x_r_* are the 90%-maximal-intensity boundaries to either side of the peak, and *f(x)* is the value of the intensity profile at point *x* along the midline.

